# Gene drives: an alternative approach to malaria control?

**DOI:** 10.1038/s41434-024-00468-8

**Published:** 2024-07-22

**Authors:** Kubendran Naidoo, Shüné V. Oliver

**Affiliations:** 1 SAMRC/Wits Antiviral Gene Therapy Research Unit, Faculty of Health Sciences, University of the Witwatersrand, Johannesburg, South Africa; 2 National Health Laboratory Service, Johannesburg, South Africa; 3 Wits Research Institute for Malaria, Faculty of Health Sciences, National Health Laboratory Service, University of the Witwatersrand, Johannesburg, South Africa; 4 Infectious Diseases and Oncology Research Institute (IDORI), Faculty of Health Sciences, University of the Witwatersrand, Johannesburg, South Africa; 5 Centre for Emerging Zoonotic and Parasitic Diseases, National Institute for Communicable Diseases of the National Health Laboratory Service, Johannesburg, South Africa

**Keywords:** Infectious diseases, Chromosomes

## Abstract

Genetic modification for the control of mosquitoes is frequently touted as a solution for a variety of vector-borne diseases. There has been some success using non-insecticidal methods like sterile or incompatible insect techniques to control arbovirus diseases. However, control by genetic modifications to reduce mosquito populations or create mosquitoes that are refractory to infection with pathogens are less developed. The advent of CRISPR-Cas9-mediated gene drives may advance this mechanism of control. In this review, use and progress of gene drives for vector control, particularly for malaria, is discussed. A brief history of population suppression and replacement gene drives in mosquitoes, rapid advancement of the field over the last decade and how genetic modification fits into the current scope of vector control are described. Mechanisms of alternative vector control by genetic modification to modulate mosquitoes’ immune responses and anti-parasite effector molecules as part of a combinational strategy to combat malaria are considered. Finally, the limitations and ethics of using gene drives for mosquito control are discussed.

## Introduction

The mosquito is one of the deadliest animals on the planet [1]. This vector has transmitted a range of pathogens that have not only had a severe impact on general human morbidity and mortality but also shaped the human genome. This includes malaria, which is arguably the most important infectious disease of the world [2]. Malaria is caused by *Plasmodium* parasites which are transmitted through the bite of an infected female *Anopheles* mosquito. As the mosquito feeds on the human host, it releases *Plasmodium* sporozoites into the peripheral circulation where they migrate to the liver. Following an exo-erythrocytic replication cycle, merozoites are released from the liver and invade red blood cells to mark the beginning of the intraerythrocytic cycle. Here, parasites undergo an asexual replication cycle which is responsible for the pathogenesis of the disease. A fraction of these undergoes a complex sexual development cycle that leads to formation of gametocytes that can be transmitted to an uninfected mosquito, completing the parasite’s life cycle [3]. It is widely acknowledged that a combined strategy of killing the parasite and limiting mosquito populations simultaneously are essential to eliminate malaria. This review focuses on vector control. There are more than 500 *Anopheles* species of which ~60 are considered important malaria vectors [4]. The use of insecticides for vector control with Indoor Residual Spraying (IRS) and distribution of Long-Lasting Insecticide-Treated Nets (LLINs) has played a significant role in reducing the burden of malaria. However, the emergence and spread of mosquito resistance to insecticides highlights the importance of developing novel insecticides and other strategies to achieve sustained control of the vector. Indeed, after more than a decade of success in the fight against malaria [5], data from 2015 onwards suggest that there has been no significant progress in reducing the global number of malaria cases and the fight against malaria will be increasingly difficult [6]. The most severe burden of malaria is localised to regions of sub-Saharan Africa, and because only a few species are responsible for transmission [4], a targeted approach to suppress local populations of the vector could have a significant impact on transmission.

## Strategies to control mosquito populations

A key element of vector control is reduction of contact with humans. This can be achieved by reducing mosquito populations and risk of the surviving vector feeding on humans [7]. Most mosquito population suppression interventions make use of insecticides to kill the adults although some programmes also incorporate larviciding [8]. Despite some success, IRS and LLINs are less effective against outdoor biting and resting females since they are designed to target indoor biting and resting mosquitoes [9]. Furthermore, widespread prevalence of insecticide resistance is a challenge to vector control [10]. As such, an integrated approach is required to expand malaria control or elimination beyond reliance on chemical interventions. Integrated pest management involves intensive surveillance to understand regional population dynamics and vector behavioural ecology. This approach requires identification of novel control technologies to achieve long-term success [11]. Some of the most promising novel interventions include housing modifications that include eave tubes [12], mosquito-attracting toxic sugar baits [11] and use of endectocides such as ivermectin to control outdoor biting mosquitoes [13–16]. These technologies are being tested in both the laboratory and field [17].

Apart from chemical intervention, the most widely used technique to control mosquitoes is the sterile insect technique (SIT). The method has been in practice since the 1950s [18] and relies on the mass rearing of males that are treated with γ-rays or chemosterilants. This leads to chromosomal aberrations and dominant lethal mutations in their sperm. After release to mate with females, sterile males do not produce offspring. Over time, the population of the targeted mosquito species in an area are reduced. However, radiation-induced damage to somatic cells can lead to reduced survival and sexual competitiveness of sterile males when compared to wild males [19, 20]. Successful suppression therefore relies on the sustained release of sterile males that outnumber wild males. SIT, coupled with surveillance, would be a useful part of an integrated vector management programme when conventional control mechanisms are challenging. Success has been demonstrated by the control of a range of agricultural pests, particularly the New World screwworm fly. SIT was originally developed for these obligate ectoparasites and was successful in eradicating the pest from North and Central America [21]. SIT has been successful against *Aedes* mosquitoes *(Ae. aegypti and Ae. albopictus)*, which spread viruses such as dengue, Zika and chikungunya [22, 23]. An example of impact is that no mosquito eggs were found in ovitraps positioned over a 50-hectare area in Havana, Cuba towards the end of a 20-week SIT trial that involved a release of 1.27 million irradiated *Ae. aegypti* males [22]. SIT however, has not been deployed for *Anopheles* control to date [24, 25]. This is due issues related to sex separation procedures, amongst others (species-specific challenges, mating behaviour and fitness of sterile males), are limiting factors that could result in the release of females which can transmit the malaria parasite [26].

SIT can be advanced by self-limiting technologies such as the release of insects with a dominant lethal (RIDL). This method relies on introduction of genetically engineered male insects that are homozygous for a repressible dominant lethal genetic trait that causes death in progeny [27]. When larvae are reared in restrictive conditions, male and female offspring do not survive to adulthood due to over-expression of a lethal effector gene. Permissible conditions are provided by adding tetracycline to the larval medium, suppressing the ‘Tet-off’ genetic system. In the wild, releasing these insects over time leads to mating between released males and wild females, resulting in population suppression as their progeny do not survive without tetracycline. RIDL does not require radiation-based sterilisation and simplifies delivery since eggs, instead of adults, can be placed at sites. Laboratory trials have demonstrated elimination of mosquito populations within 20 weeks and highlight the potential application of RIDL in vector suppression [28]. Female specific (fsRIDL) is a variant of RIDL that specifically targets female mosquitoes. This involves a repressible flightless female phenotype where the daughters of released male mosquitoes with the RIDL trait inherit the flightless phenotype. Systems have been developed for *Ae. aegypti*, *Ae. albopictus* [29] and *An. stephensi* [30]. Advantages of fsRIDL include an automatic sexing system where the female-specific lethality allow males to pass on the gene to future generations, albeit it with decreasing effect with each subsequent generation. Despite promise, there are problems due to a high fitness cost to males. Genetic modification has also been used for precision-guided SIT in *Ae. aegypti*. Flightless females and sterile males can be generated by employing clustered regularly interspaced short palindromic repeats (CRISPR) and Cas-associated gene editing technology. This non-gene drive genetic modification can be used to suppress populations in a confinable and reversable manner [31]. Genetic modification has thus expanded the utility and efficacy of SIT.

The relatively recent sequencing of the *Anopheles* genome [32] and significant advancements in gene editing tools have made it possible to create genetically modified mosquitoes (GMMs) that carry a self-limiting gene. This form of genetic control advances SIT beyond simple sterilisation and enables passing on of traits that may be useful for vector control [33]. The annotated mosquito genome also allows for identification and targeting of genes that are involved in reproduction [34], key genes involved in host-seeking behaviour [35, 36] and susceptibility to parasite infection [37, 38]. Frequencies of such modifications are however, expected to decrease with time since males cannot reproduce and the approach will require periodic batch releases of GMMs. Several GMM lines with variable resistance to *Plasmodium* have been developed [39]. However, they are not part of malaria control programmes. Factors that need to be considered before implementation of these organisms include incomplete resistance, community acceptance of GMMs and implications of negative fitness effects. Lower fitness will limit ability of mosquitoes to spread traits through populations and compete with the wild insects [40, 41].

A recently developed strategy involves use of the bacterial symbiont, *Wolbachia*. This Gram-negative bacterium is found naturally in many insects [42] and high densities in insect eggs results in vertical transmission. Infection by *Wolbachia* induces a range of sex distortion phenotypes. This includes feminisation, male killing, parthenogenesis and cytoplasmic incompatibility (CI). Use of *Wolbachia* may be thought of a type of gene drive as it is inherited in a non-Mendelian manner. However, since it is not due to direct genetic manipulation, it is a paratransgenesis intervention. *Wolbachia* makes use of incompatible males to cause sterility or introduce deleterious fitness effects to reduce vector competence. The first involves CI, which prevents eggs of uninfected females from hatching when fertilised by infected males. It is negated by males mating with infected females. This could be exploited in SIT applications in a method referred to as Incompatible Insect Technique [43]. The second strategy impairs vector competence, where certain strains of *Wolbachia* reduce the susceptibility of insects to some pathogens [44]. The World Mosquito Programme is currently using *Wolbachia* to control arboviruses transmitted by *Ae. aegypti* [45]. The method, however, has been less successful for malaria control. Reasons include that *Wolbachia* is not as prevalent in *Anopheles* compared to other mosquito species [46]. However, *An. gambiae* infected with natural *Wolbachia* can reduce *Plasmodium* development [47]. At present, it is unclear whether *Wolbachia* induces CI in *Anopheles*. Furthermore, it is hypothesised that Anopheline symbionts and immunity may limit *Wolbachia* infection [48]. There are still more factors that need to be understood, but at present, the low densities of the symbiont in *Anopheles*, potential lack of CI [46] and the lack of vertical transmission [48] makes *Wolbachia* a poor candidate for malaria control. *Wolbachia* was thought to be absent in *Anopheles* and *Ae. aegypti* mosquitoes and their recent detection in those species [49, 50] has led to studies on the mosquito and role in pathogen suppression. Their long-term impact on mosquito populations and disease transmission requires investigation.

Success of SIT and GMMs is dependent on several factors. These include technical capacity, capital investment, the size of the affected areas, migration patterns of mosquitoes and ability to produce and release mosquitoes in endemic areas [51, 52]. Most of the global malaria burden is localised to sub-Saharan Africa [53], where vectors are distributed across large resource-limited rural areas. It may not be feasible to release the required number of SIT and GMM to make a significant impact. If possible, it is likely that the effect of SIT will be diluted and GMM traits will be lost relatively quickly [54]. Therefore, methods that allow for a selection bias in the spread of a genetic trait are not only desirable but necessary to limit the spread of mosquito-borne diseases.

## Genetic control of mosquitoes using gene drives

**Fig. 1:**
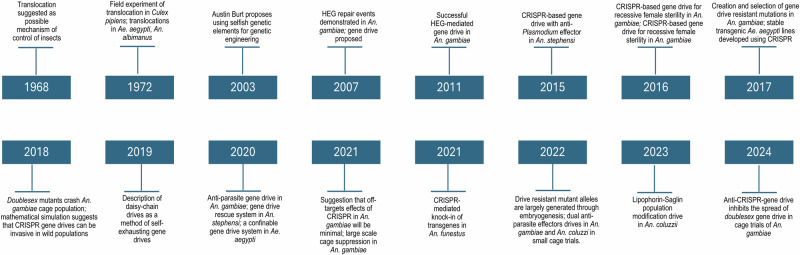
Timeline of key events in the development of gene drive systems in mosquitoes [55, 58, 65, 76, 87, 123, 131–138]. Image created with BioRender.com.

A strategy that has gained momentum in recent times is the direct introduction of genetic traits that spread rapidly through a mosquito population and impact their ability to transmit disease. A genetic manipulation may be introduced to suppress mosquito reproduction [55] or inhibit pathogen development within the vector [56]. A gene drive is a natural process that is based on preferential inheritance. Here, a gene is passed on from parent to offspring at a greater than Mendelian rate. This bias allows the gene to spread through a population within a few generations [57]. The possibility of creating gene drives was introduced in 2003 [58], when selfish genetic elements were observed to copy themselves into a specific target DNA sequence. These homing endonuclease genes (HEGs) encode DNA endonucleases that recognise and cut, in chromosomes that do not contain the HEG, at the very site in the genome where they are inserted. In this way, the cleaved chromosome will be repaired using the HEG-containing homologue as a template, converting the HEG from heterozygosity to homozygosity and thus, enable genome editing. The characterisation and rapid development of CRISPR has made it easier to re-programme endonucleases to recognise a target sequence of choice. HEG and CRISPR-Cas9 are the two most developed gene drive systems and a timeline with key findings that led to establishment of methods for vector control is summarised in Fig. 1.

**Fig. 2:**
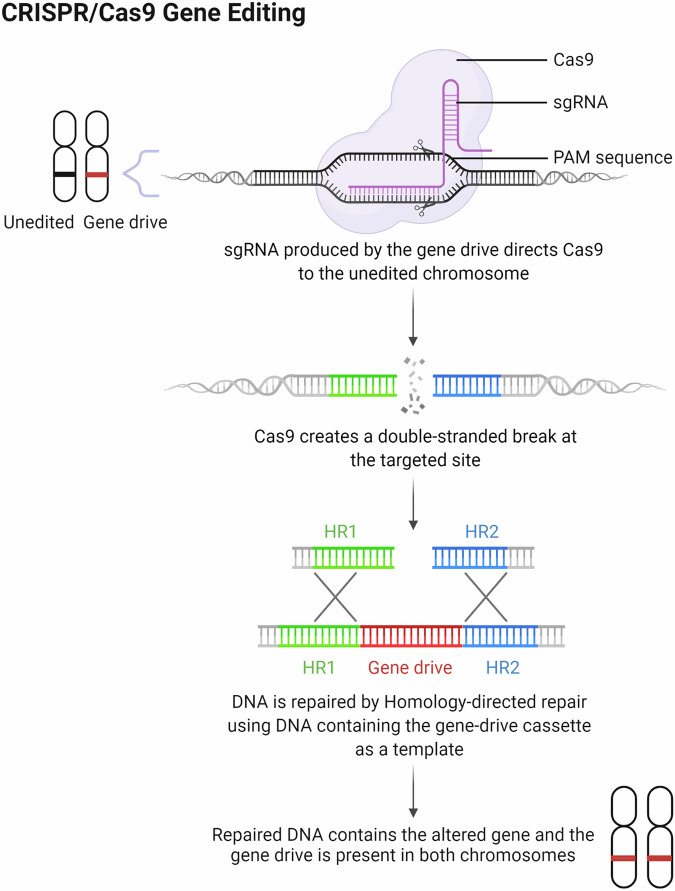
Creating a CRISPR-based gene drive. The sgRNA binds Cas9 when expressed in the chromosome containing the gene drive cassette. Cas9 is then directed to bind and cleave DNA at complementary sites, usually 20 nucleotides in length, on the unmodified chromosome. The cassette encoding the sgRNA and Cas9 sequences is flanked by homologous sequences which allows for homology-directed DNA repair and duplication of the gene drive cassette. The gene drive may then be passed to almost all the progeny and can modify the population within a few generations. HR homologous region, PAM protospacer adjacent motif, sgRNA single guide RNA. Image created with BioRender.com.

The technology, in the case of CRISPR-Cas, can be applied to diploid organisms such that the gene drive cassette containing a Cas9 gene is inserted into a chromosome (Fig. 2). The cassette is under the control of a germline-specific promoter and includes a single guide RNA (sgRNA). The sgRNA directs the Cas9 endonuclease to a specific recognition site of the unmodified chromosome. This recognition site is lost in the modified chromosome. When paired with the wild-type chromosome, the sgRNA directs the Cas9 protein to the target sequence to create a double-stranded (ds) DNA break. Sequences flanking the DNA break are homologous to the regions flanking the chromosome with the gene drive. This serves as a template to repair the DNA break using the host’s homology-directed DNA repair process. The gene drive cassette is therefore duplicated and present in both chromosomes.

**Fig. 3:**
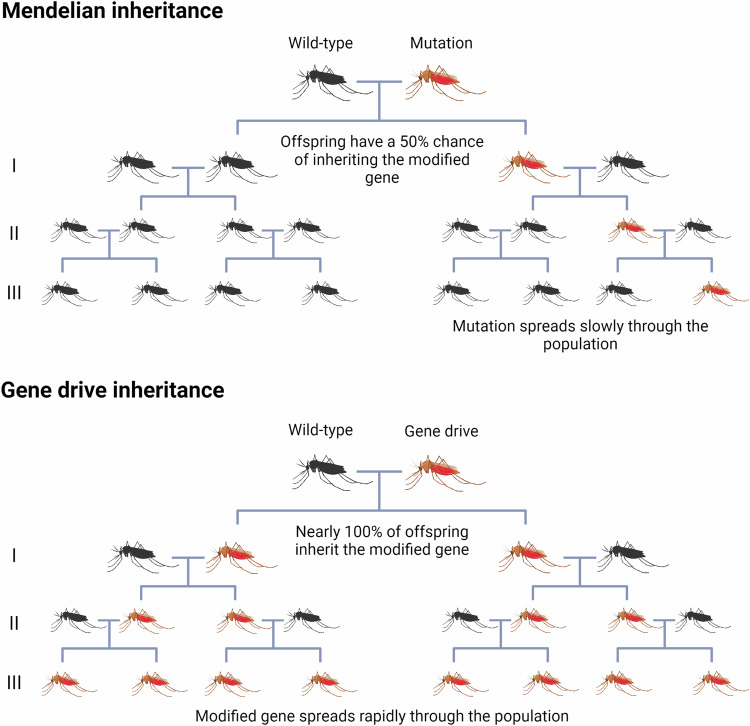
Comparison of Mendelian versus gene drive inheritance patterns. In each case, a few transgenic mosquitoes (red) are introduced into a large wild-type population (black). Gene drives allow the desired trait to spread through a population more quickly. Image created with BioRender.com.

Usually, a genetic change in one organism takes a long time to spread through a population because a mutation carried on one of a pair of chromosomes is inherited by only half the offspring. By homing, a proportion of germline cells will be converted to homozygosity and thus, a higher proportion of the gametes from heterozygous mosquitoes will contain a copy of the gene than would be expected for normal Mendelian inheritance. Therefore, gene drives can rapidly increase in frequency with each generation (Fig. 3). Success has been demonstrated in *Anopheles* mosquitoes, where gene drives targeting the *doublesex* fertility gene in *An. gambiae* had an inheritance bias of 100% [55] and female mosquitoes could not bite or lay eggs after ~10 generations. Since the gene is highly conserved in *An. gambiae* [59] and critical for breeding, the gene is less likely to retain resistant mutations [60]. In this case, the gene drive was used to disrupt an essential gene in the mosquito such that recessive heterozygote mosquitoes, but not homozygous insects, are viable. The mosquito population is reduced when the homozygous disrupted gene is inherited.

## Suppression vs modification gene drives

**Fig. 4:**
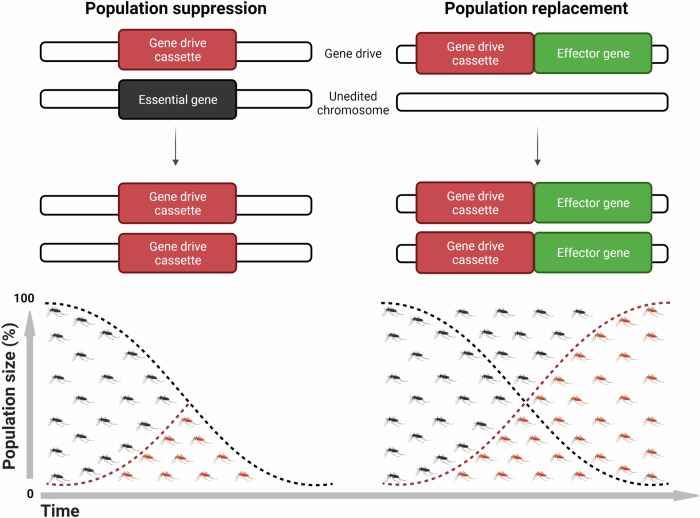
Gene drive population suppression vs replacement strategies. A modified mosquito (red) containing a gene drive which disrupts an essential gene to produce a desirable phenotype, such as female infertility, can reduce a wild-type (black) and total mosquito populations over time. An important characteristic is that heterozygous mosquitos should be viable to allow them to pass on the gene drive to the next generation. Alternatively, gene drives may be engineered to contain an effector molecule which can, for example, become refractory to the malaria parasite. These modified mosquitoes may be used to invade wild-type populations and replace malaria-susceptible mosquitoes. Image created with BioRender.com.

Regardless of the mechanism used to drive a gene through a population or disrupt an important gene, there are two strategies for vector control. The first is population suppression, where an essential gene involved in mosquito reproduction is targeted to potentially eliminate a population (Fig. 4). This may be considered as the genetic equivalent to use of insecticides and an example is the gene drive targeting the *doublesex* gene. The second strategy is population replacement, where the population is modified to drive a trait that prevents transmission of a pathogen [61]. This can be considered as immunising the population. Examples includes single-chain antibodies or exogenous antimicrobial peptides that are attached as cargo. Therefore, suppression reduces vectorial capacity, while replacement reduces infectivity.

The frequency of gene drive-related studies has increased significantly in recent times. Table 1 highlights recent progress in mosquito gene drive technology. This includes identifying gene drive candidates that meet certain criteria such as drive efficiency, effector efficacy, genetic fitness, safety, release strategies and minimal essential requirements for a practical product. For example, AgNosCd-1 targets the *cardinal* locus which produces haem peroxidase in *An. gambiae*. It causes the red-eye mosquito phenotype and has a drive efficiency of 98–100% in both sexes. Single releases of AgNosCd-1 males at equal transgenic to wild-type ratios achieved full introduction of the gene drive in small cage laboratory trials within six generations. Importantly, the drive had no major impact on fitness, off-target effects and resistant alleles emerged at a low frequency [62]. This drive is being explored as a vehicle to deliver anti-*Plasmodium* effector molecules across modified mosquito field strains.

**Table 1:** Summary of recent gene drive suppression or replacement studies.

Year (Ref)	Species	Gene drive system	Effect
2008 [120]	*An. gambiae*	HEG-based: I-pPol endonuclease expression in 28 S ribosomal genes in X Chromosomes carrying sperm.	Suppression: Heterozygous males crossed with WT females have dominant early embryo lethality.
2011 [121]	*An. gambiae*	HEG-based: biased expression of I-Sce1 endonuclease, with proof that gene drives can invade caged populations.	Suppression/replacement: Validation of modelled HEG transmission dynamics for population-level genetic engineering.
2014 [122]	*An. gambiae*	HEG-based: biased expression of I-pPol endonuclease in X Chromosomes.	Suppression: Male sterility.
2015 [123]	*An. stephensi*	CRISPR-Cas9-based: AsMCRkh2 construct targeting the *kynurenine hydroxylase* white locus (ASTEI06357).	Replacement: Introduction of single chain m2A10-m1C3 antibodies for Anti-*Plasmodium* activity.
2016 [34]	*An. gambiae*	CRISPR-Cas9-based: First and second-generation gene drive strains targeting the genes AGAP005958, AGAP011377 and AGAP007280.	Suppression: Recessive females’ sterility with the gene AGAP007280 meeting minimum gene drive requirements.
2017 [124]	*Ae. aegypti*	CRISPR-Cas9-based: Disruption of the *kynurenine hydroxylase* gene (AAEL008879).	Suppression/replacement: Generation of multiple stable transgenic lines.
2018 [55]	*An. gambiae*	CRISPR-Cas9-based: Disruption of the d*oublesex* (Agdsx) splice variant, dsx-female (AgdsxF).	Suppression: Progressive reduction of egg production due to sterile intersex females.
2018 [125]	*An. albimanus, An. gambiae, An. coluzzii, An. funestus*	CRISPR-Cas9-based: Disruption of the *White protein* gene (ACOM037804).	Suppression/replacement: Successful application of CRISPR-Cas9 mutagenesis in a range of diverse species.
2019 [126]	*An. stephensi*	CRISPR-Cas9-based: Development of the transgenic AsMCRkh2 gene drive strain expressing the m1C3 and 2A10 single chain antibodies.	Replacement: Anti-*Plasmodium* genes are spread more efficiently using gene drive systems. However, this comes at a fitness cost to the mosquito.
2020 [62]	*An. gambiae*	CRISPR-Cas9-based: Development of the *AgNosCd-1* strain developed using the pCO37 construct targeting the Haem peroxidase 6 *AgHPX6* (AGAP003502).	Replacement: Development of gene drive strain for population modification without significant fitness effects and low frequency of resistance alleles.
2020 [127]	*An. stephensi*	CRISPR-Cas9 based: Development of VgCp26.10 transgenic strain which targets the Enhanced cyan fluorescence protein, ECFP.	Suppression/replacement: Successful gene editing using Receptor-Mediated Ovary Transduction of Cargo (ReMOT) control as delivery method, as opposed to embryo injection.
2020 [128]	*Ae. aegypti*	CRISPR-Cas9 based: Disruption of AAEL016999, an ATP binding gene.	Replacement: Development of split gene drive systems which aid the development of effector-linked gene drives.
2020 [129]	*Ae. aegypti*	CRISPR-Cas9 based: Disruption of female specific transcripts of Actin (*AeAct-4*) and myosin (*myo-fem*).	Suppression: Generation of flightless females. Two intact copies of *myo-fem* are required for flight.
2021 [107]	*An. gambiae*	CRISPR-Cas9 based: Modification of the Zinc carboxypeptidase A1 (CP; (AGAP009593), peritrophin1 (Aper1; AGAP006795), alkaline phosphatase 2 (AGAP006400) genes.	Replacement: Creation of non-autonomous gene drives by minimal changes that also allow the expression of an effector gene. The process was termed ‘integral gene drive’.
2021 [130]	*Cx. quinquefasciatus*	CRISPR-Cas9 based: Creation of a AGG2069 *kmo* HDR donor construct targeting kynurenine 3-monooxygenase (CPIJ07147).	Suppression/replacement: Proof of concept for the expression in vivo of sgRNA after a knock-in cassette in this species.
2021 [131]	*An. funestus*	CRISPR-Cas 9 based: vasa2: SpCas9 construct targeting *An. funestus white* gene (AFUN003538).	Suppression/replacement: Germline delivery of CRISPR components in *An. funestus*.
2022 [88]	*An. gambiae*	CRISPR-Cas 9 based: Two midgut host genes of *An. gambiae* to co-express magainin 2 and melittin.	Replacement: Impairment of oocyst development in both *P. berghe*i and *P. falciparum*.
2023 [132]	*An. gambiae*	CRISPR-Cas 9 based: Lipophorin gene in *An. gambiae* to co-express magainin 2 and melittin.	Replacement: Impairment of oocyst development in both *P. berghe*i and *P. falciparum*.
2024 [76]	*An. gambiae*	CRISPR-Cas 9 based: Transgenic *An. gambiae* expressing anti-Cas AcrIIA4 protein.	Reversal of gene drive: First successful test of an anti-drive system in large cages.

There are advantages and disadvantages to both types of drive systems. Population modification through replacement drives is predicted to remain stable for longer periods of time to allow for localised elimination of a pathogen. This could be scaled up to allow for potential elimination of a pathogen across large regions and is an advantage over a suppression approach. Like insecticides, suppression drives may need to be applied repeatedly in the same area [63]. However, replacement drives do not allow for niche replacement where a major vector is eliminated [64]. Therefore, they are likely to have less of an ecological effect. Despite advantages, development of replacement drives has lagged compared to suppression drives. This is due to challenges including anti-pathogen cassettes which would need to be developed for each pathogen. For example, in regions where multiple malaria parasites, arboviruses or mosquito species circulate. Here, redundant effectors would be needed to avoid the selection of resistance to the effector.

Suppression drives are more prone to the greater effects due to errors in copying. This is due to their intended design, which disrupts genes rather than delivering a cargo. Despite this disadvantage, it is likely that suppression drives will be deployed first since it is more complex to develop replacement drives. Although suppression drives suffer due to small mutations, most non-homologous end-joining (NHEJ) mutations are non-functional and therefore, passively contribute to population suppression [65]. An example of this is an NHEJ that results in a non-functional protein, that although inhibits the gene drive still is deleterious to protein function. This impairment of the function of the putative target therefore serves as a passive mechanism to suppress the population [65]. The *doublesex* suppression drive, however, has not developed functional drive-resistant alleles.

## Inhibiting parasite development in mosquitoes

**Fig. 5:**
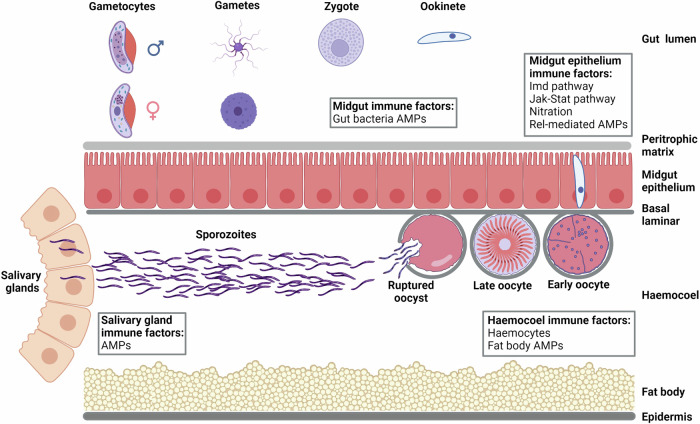
Stages of potential genetic intervention during the life cycle of the malaria parasite in the mosquito. Simplified schematic highlighting the physical and physiological barriers in the mosquito, including the midgut epithelium, haemocoel and salivary glands, that *Plasmodium* parasites must overcome to establish infection. The types of immune response that are induced to kill parasites either by lysis, melanisation, or phagocytosis, are boxed. Parasite numbers are at their lowest in the midgut and many ookinetes do not survive the immune response as they traverse the midgut epithelium. These stages are attractive targets for strategies aimed at stopping parasite development. Gene drives that can modify the vectors’ immune system to neutralise parasites in the midgut could reduce disease transmission. AMPs anti-microbial peptides. Image created with BioRender.com.

Since population modification strategies are less prone to developing resistance, population modification strategies do not risk the creation of new vector niches or alter the trophic structure of the areas. They may be an attractive option for gene drives. However, finding suitable biological targets to introduce into a population is a challenge. Possible targets include the mosquito’s immune system [66]. Female mosquitoes have a range of immune barriers and defences to protect against pathogens during feeding (Fig. 5). Some of the most basic defences are epithelial barriers that need to be traversed by the parasite. Upon a blood meal, the mosquito rapidly develops a peritrophic matrix, a chitinous membrane that encases the blood meal to protect the gut from blood-borne challenges. The midgut represents the first challenge for any pathogen [67]. It is also the first of three major immunological compartments that the pathogen encounters; the others being the haemocoel and salivary glands. Pathogen killing in each compartment is mediated by one of three major mechanisms: cell-mediated phagocytosis, melanisation and lysis. The factors that lead to killing can be either cellular, which include phagocytosis, or humoral such as melanisation or the production of antimicrobial peptides [68].

**Fig. 6:**
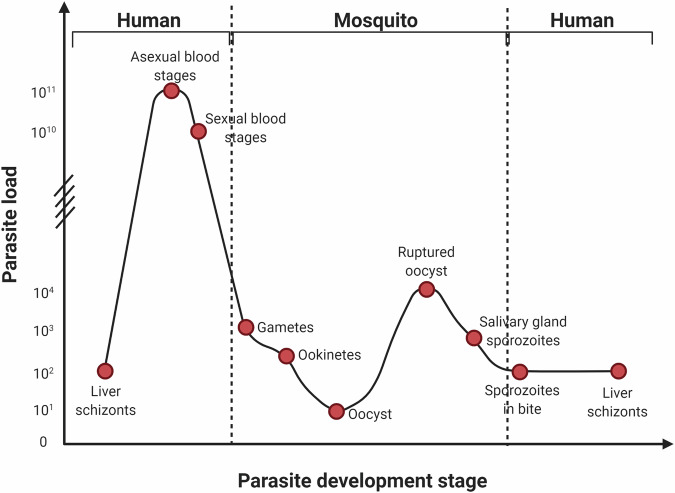
Population of *Plasmodium* parasites at each developmental stage. The number of malaria parasites during growth and proliferation within each host, which range from less than 10 ookinetes in the mosquito to trillions of blood stage parasites in the human [97]. Image created with BioRender.com.

In the midgut, the native gut microflora as well the blood-borne proteins induce production of numerous immune effectors. This results in proliferation of haemocytes and activation of immune signalling pathways. In the case of *Plasmodium* infection, the most potent response is initiated by the immune deficiency (IMD) pathway [68, 69]. The Janus kinase-signal transducer and activator of transcription pathway regulates Nitric Oxide Synthase activity and is a key factor in the control of oocysts [68]. During a *Plasmodium* infection, gametocytes reproduce sexually to form ookinetes, which is the stage of the *Plasmodium* life cycle with the lowest number of parasites (Fig. 6). Ookinetes traverse the midgut as they move towards the basal lamina. Their presence initiates a complement-like cascade, which can reduce the number of ookinetes by up to 10,000-fold. However, only a few viable ookinetes are sufficient to continue with the parasite’s development. Oocysts must survive the haemocoel compartment as they develop into sporozoites, which invade the salivary glands to complete the parasite’s developmental cycle in the mosquito [70]. Oocysts are exposed to a range of humoral immune factors in the haemocoel and salivary glands also produce antimicrobial proteins, although the immunological role of the latter is unclear [68]. The immunological response to the parasite is largely effective, but complete refractoriness and inability to transmit the parasite are rare in nature. This is possibly a result of fitness costs of immunological defences [71]. There are evolutionary advantages to the preservation of vectorial capacity [72]. This could underlie the difficulty in immune modulation of the mosquitoes for vector control. Despite these challenges, effective immune modulation or modification of mosquitoes could result in blocking parasite transmission, making it an attractive option for malaria control.

## Controlling gene drives

Regardless of the gene drive target or strategy, a successful programme may affect the environment where it would be deployed. The potential to change a natural population permanently raises concerns about unintended effects of gene-driven transgenes. Strategies to limit and regulate the long-term effects of the gene drive may be useful. Two HDR-mediated systems that may neutralise gene drives have been proposed. These are based on conditional drive systems that encode gRNAs, without the Cas9 gene: erasing construct hitchhiking on the autocatalytic chain reaction (e-CHACRs) and delete elements reversing the autocatalytic chain reaction (ERACRs) [73]. eCHACRs act by carrying one gRNA to copy themselves at their site of genomic insertion, as well as additional gRNA(s) to mutate and inactivate Cas9 produced in trans by the gene drive. Proof of principle has been demonstrated in *Drosophila melanogaster*, where e-CHACRs were efficient (>99%) in mutating and inactivating Cas9 with different gRNAs targeting the *Cas9* gene within 10 generations [73]. The inactivated drive, however, remains in the genome. ERACRs on the other hand, are inserted into the genome at the same site as a gene drive. They contain two gRNAs to direct cleavage on either side of the gene drive and replace the gene drive cassette with ERACR sequences. ERACRs can occasionally recombine with gene drive sequences due to short stretches of sequence complementarity. This can create chimeric ERACR-drive elements that retain the gene drive activity. This limitation can be improved by eliminating homology between ERACRs and the gene drive. However, ERACRs usually restore the activity of a gene disrupted by the gene drive in population cage studies [73]. Cas9-triggered chain ablation (CATCHA) [74] is another drive-neutralising element and is similar in design to e-CHACR and ERACR. It carries a gRNA that targets Cas9 for mutagenesis (like e-CHACR) but is inserted into the same genomic site as the gene drive (like ERACR). eCHACRs, ERACRs and CATCHA have potential to halt or delete a gene drive at growth dynamics similar to gene drive trajectories in wild-type populations [73]. However, their performance in mosquitoes requires evaluation. It may be possible to combine these various drive-neutralising approaches with elements carrying anti-Cas9 proteins [75]. Indeed, an anti-Cas9 transgene inherited in a Mendelian fashion can neutralise the activity of the suppression *doublesex*-drive [75], including in large cages which contained age-structured overlapping populations that exhibited more complex behaviour and natural ecological conditions [76]. Theoretical strategies to split a gene drive into multiple pieces, such as the daisy-chain gene drive systems [77], could limit the spatial distribution of transgenes. In this case, the DNA components that make up the gene drive cassette are split and scattered around the organism’s genome such that none can singly drive the gene. However, the components are connected such that the organism with the first element drives the second which, in turn, drives the third element and eventually leads to spread through the species. This may not prevent long-term establishment of one or more transgenes in nature. Although potential hazards of possible DNA rearrangements are unknown, the strategy allows a local drive system to persist for a few generations before daisy elements are lost and the drive becomes redundant in a localised area [77]. Other potential mechanisms of self-exhausting or self-limiting strategies include use of site-specific recombinases, transposases-mediated excision as well as single-strand annealing-based DNA repair to excise the gene drive cassette and revert the modified chromosome to a non-transgene allele. Models that calculate the dynamics of spread and persistence of gene drive transgenes critical for female fertility in the presence of self-elimination mechanisms show that this approach can provide temporal control and reverse invasion of a gene drive transgene, even at low rates of effectiveness (less than 10%), while tolerating substantial rates of failure [78]. These suggest it is possible to create gene drives that may be removed from the environment.

## Future perspectives for utilisation of gene drives

Mosquito control using genetic modification is gaining momentum. The release of a set of guidelines by the WHO, with a special focus on ethics and safety of GMM and gene drives, supports its application [52]. Non-gene drive mechanisms have successfully been deployed in the field for non-anopheline mosquitoes, such as modified *Ae. aegypti* in Brazil and Malaysia [79, 80] and are being tested in malaria vectors. The *An. coluzzii* dominant Sterile Male strain Ac(DSM)2 for example, carries a HEG that causes complete sexual sterility in male carriers by disrupting the X-chromosome in spermatozoa. The Target Malaria consortium performed the first field release of this genetically modified species in Burkina Faso [81] to assess the potential fitness cost and gain insight into the dynamics of transgene-carrying mosquitoes. Efforts to investigate gene drives in Africa and on malaria vectors is a positive move.

Genetically modified *Ae. aegypti* have been released in several sites to reduce the number of these disease carrying mosquitoes. Examples include the OX513A strain that was released in the Cayman Islands that led to an 80% suppression of the *Ae. aegypti* strain [27], as well as the OX5034 strain which was released in Florida in 2021 [82, 83]. GMMs remain a subject of debate, however they can potentially impact on disease prevention and mosquito population control. They are being closely monitored by scientists and regulatory authorities.

Although RIDL or *Wolbachia*-based mechanisms are not in use for *Anopheles* mosquitoes, large cage trial successes in population suppression using *doublesex*-based gene drive are promising [84]. RIDL technologies are being developed against *An. stephensi*, which has recently invaded the Horn of Africa. Unlike most malaria vectors, it is adept at colonising urban environments. Therefore, its arrival in Africa raises the threat of increased malaria in cities that it invades [85]. Female specific flightless *An. stephensi* have been developed for the release of males only by companies such as Oxitec as an option to control the vector.

Successful population replacement gene drives have been demonstrated in the laboratory and small cages [62, 86], but these drive systems are not as advanced as that of population suppression. Recent successes in replacement drives, such as the expression of single chain monoclonal antibodies against both sporozoites and ookinetes in both *An. gambiae* (AgTP13) and *An. coluzzii* (AcTP13), demonstrates its potential impact. Here, gene drives achieved full introduction within 6 months after release in small cage trials and effector molecules reduced both parasite prevalence and infection intensities, with no fitness loads affecting AcTP13 (reduced for AgTP13) gene drive dynamics when compared to wild type mosquitoes [87]. Replacement gene drives in *An. gambiae* are also being tested in Tanzania [88], where a genomic locus was modified to express two exogenous antimicrobial peptides (magainin 2 and melittin) which impeded the transmission of *P. falciparum* and *P. berghei* malaria parasites, as well as reduced the lifespan of the mosquito. The immune system is a promising target for replacement drives. Augmentation of the immune system would reduce mosquitoes’ transmission of the parasite, and this has previously been demonstrated [89]. However, refractory strains may suffer from fitness costs of increased production of immune factors, antimicrobial peptides [90], factors that opsonise parasites [91] or high expression of the IMD promoter *REL2* [92]. All these factors may shorten mosquitoes’ lifespan.

Less explored options for improving mosquitoes’ responses to the parasite include genome incorporation of sequences encoding anti-*Plasmodium* effectors, such as single chain antibodies [51] or anti-microbial peptides [93, 94]. Transmission could also be reduced by human vaccines. The pre-erythrocytic circumsporozoite vaccine, R21, demonstrated a promising 77% efficacy a year after trial administration [95] and has since been become the first parasite vaccine to receive regulatory approval [96]. These are in addition to the transmission blocking vaccine (TBVs) strategies that are being developed as part of a concerted effort to target various stages of parasite development simultaneously. The rationale for the approach is that the production of antibodies against gametes in the vertebrate host, which are transmitted to the mosquito during a blood meal, prevent insects from spreading disease [97]. Several studies have demonstrated feasibility of this strategy. For example, a complete block in transmission of *P. falciparum* from field isolates was achieved when incubated with antibodies against the mosquito-stage gametocyte antigens, Pfs230, Pfs48/45 and Pfs25 [98]. Transmission reducing activity was also achieved in preclinical studies [99]. The surface protein, Pfs47, allows *P. falciparum* parasites to evade the mosquito’s immune system in the midgut [100]. Pfs47 is expressed on the surface of female gametocytes, zygotes and ookinetes and allows ookinetes to bind to the midgut receptor before the parasite traverses the midgut epithelium. This is a critical phase as the parasite load is relatively low at this stage and most ookinetes are eliminated by the mosquito’s immune system when they traverse the midgut. Antibodies against a 52 amino acid central region of Pfs47, however, reduced transmission by up to 99% [101]. TBVs have not been explored as gene drive effector molecules. However, combining strategies such as gene drives and TBV that share a common target is likely to have a greater impact on eliminating the transmission of malaria.

An alternative population replacement strategy is use of gene drives to disrupt genes that promote parasite development. This would reduce the capacity to transmit the parasite and shift the population towards lower vector competence. Such targets include the C-type lectin 4 (CTL4) and CTL mannose binding 2 (CTLMA2) proteins. These lectins are believed to bind to the *O*-GlcNAcylations on glycoproteins expressed on ookinetes [102]. Knockdown of these genes results in increased parasite melanisation and a reduction in oocyst burden [103]. This reduces or eliminates the amount of sporozoites transmitted, thereby interrupting the transmission cycle. The enzyme, Immunomodulatory peroxidase (IMPer), is part of a peroxidase/dual oxidase system that regulates epithelial immunity in the *An. gambiae* midgut. This system plays a role in mosquito susceptibility to *Plasmodium* invasion. Silencing of IMPer results in a reduction of parasite burden without affecting mosquito longevity [104], making IMPer an attractive immunomodulatory gene drive strategy. Despite the potential of targeting these genes, the effects of knockdowns and interplay with the environment require careful assessment [105]. Climate for example, can alter the general physiology of the mosquito and pesticides could select against the vector if an insecticide-susceptible strain was used to disseminate the gene drive. Therefore, not only is the evaluation of these systems complex but may be altered by the environment into which the mosquito is released. Nevertheless, immunomodulatory targets are attractive and could expand the repertoire of population replacement gene drives.

It may be useful to target genes expressed in the gut and salivary glands. This is because numbers of parasites located at these sites are relatively low in the malaria life cycle and should improve success [106]. Recent advances have been made with this approach, where gut-expressed genes were converted to non-autonomous HEGs [107]. This is known as an integral gene drive system and requires minimal genetic modification to allow integration into any host gene. As a result, endogenous genes are converted to gene drives without disrupting the function of the gene. This system also appears to reduce establishment of drive-resistant alleles in the population [107]. As such, an integrated gene drive is a useful tool for replacement drives without requiring effector molecules. More endogenous genes amenable to this system needs to be found, with the mosquito gut and salivary glands offering attractive potential targets. Although suppression drives may initially be appealing and potentially better accepted by communities, replacement drives may be better long-term alternatives to avoid problems of ecological niches being filled by minor or alternate vectors.

## Resistance to gene drives and methods of mitigation

The gene drive mechanisms described in this review have largely been studied in Phase 1 laboratory and cage studies. Possibly the greatest technical concern regarding the use of the systems is its development. Careful consideration, underpinned by mathematical modelling, of the effect of resistance mutations are critical safety concerns. Resistance to gene drives can occur through three major mechanisms. Mechanistic resistance occurs when the drive mechanism is impaired in the individual, which results in interference by causing target changes. Mechanistic resistance usually occurs due to a mutation at the endonuclease cleavage site. This can be induced by NHEJ repair or individual genetic variation [108], where selection pressure for drive resistance is at its highest. The drive can also be disrupted by selection pressure on unlinked loci. Resistance to natural and synthetic drives can occur slightly differently. Naturally occurring homing endonucleases can tolerate some changes in the individual sequences. By contrast near perfect sequence fidelity is required for CRISPR-Cas9 cleavage. As such, resistance can develop at a much faster rate than for homing endonucleases. A potentially important negator for resistance to CRISPR-based gene drives is the inclusion of multiple target sites. This can be done by targeting evolutionarily conserved sites. An example is the highly conserved *doublesex* gene, where no selection for resistance alleles have been reported. Compensatory resistance occurs when mechanisms elsewhere in the genome negates the fitness effect of drive. This can occur despite the drive evolving normally and can be considered as developing a tolerance to the drive. An example of this is an increased investment into testes in response to a loss in sperm [109]. Finally, the population structure can change, which prevents the drive from spreading through the population or becoming fixed [110]. This includes changes such as a shift to sib mating. [111].

By 2018, all CRISPR-based homing drives in insects had produced resistance alleles [112]. Mutations in the target site rendering it undetectable to the drive is a common problem in gene CRISPR-Cas9-based drives. Unlike homing endonucleases, this system requires near-perfect matching of the sgRNA and the target site. Functional resistance can develop quickly if single targets are used for CRISPR-based interventions [113]. Therefore, the use of multiple targets through multiple gRNAs, is important to prevent the development of resistance multiple guide RNAs [114]. Work on the *doublesex* gene, however, demonstrates the efficacy of targeting a highly conserved region. This strategy tends to reduce the chance of incurring mutations that would render the target site insensitive [55].

Selection of appropriate promoters can reduce the development of resistance. Germline promoters like *nanos* and *zpg* reduces the number of NHEJ repair of dsDNA breaks in alleles generated [115]. NHEJ repair tends to be non-specific and often results in mutations that render them insensitive to the gene drive. Drives inserted into the *cardinal* locus of *An. gambiae* for example, have very few non-NHEJ alleles and little fitness costs [116].

Gene drives carry safety concerns, which may be categorised as health and environmental. Direct health implications relate to unexpected effects of gene drives on the biology of the mosquito, even against the backdrop of reduced disease burden. The insecticide resistance profile of the modified mosquito population is also a consideration. Release of insecticide resistant mosquitoes is problematic because control of these insect populations by conventional methods are difficult and resistant mosquitoes may be associated with higher rates of malaria transmission [117, 118]. The efficacy of insecticide-susceptible gene drive mosquitoes could also be diluted if released into natural populations carrying high levels of insecticide resistance and where conventional control methods are used [119]. Environmental safety concerns are related to the effect of the release of GMMs on local ecosystems. This includes the transfer of the construct to related species (vertical transfer) or to unrelated species (horizontal transfer). Potential effects on the ecosystem resulting from removal of a species or the increase of detrimental competitor species are worth considering. This is true where more than one species exists in sympatry. Removal of a major vector opens a niche for secondary vectors [64]. There are other factors pertinent to implementing gene drives as a mode of malaria control that are not discussed in this review. These include ethical considerations, technology regulation, social responsibility, political support and sustainable funding.

## Summary

Targeting mosquito vector populations to interrupt disease transmission, such as malaria, are vital to successes against the disease. Recent progress with the use of gene drives has been remarkable. The successful deployment of gene drives has been proven in yeast, fruit flies and two species of mosquitoes. If used wisely, gene drives could benefit both human health and the environment. Current evidence suggests a range of advantages to replacement gene drives. The first gene drives are likely to be aimed at population suppression drive using a *doublesex*-based system in an environment supporting a single dominant vector species. However, its true impact will be evident once the system has been implemented on a large scale and evaluated in terms of how the transgene spreads across mosquito populations in the natural environment and whether it decreases mosquito populations. Gene drives are a promising new mode of disease control. However, their sole use may not be sufficient to eliminate malaria or other diseases. Gene drives are more likely to achieve success when used in combination with other vector control measures, such as surveillance, insecticide deployments, transmission blocking strategies and human vaccination to eliminate or possibly, eradicate the disease.
